# Micro-Raman Spectroscopy for Monitoring Changes in Periodontal Ligaments and Gingival Crevicular Fluid

**DOI:** 10.3390/s141222552

**Published:** 2014-11-27

**Authors:** Carlo Camerlingo, Fabrizia d'Apuzzo, Vincenzo Grassia, Letizia Perillo, Maria Lepore

**Affiliations:** 1 CNR-SPIN, Istituto Superconduttori, Materiali Innovativi e Dispositivi, via Campi Flegrei 34, Pozzuoli 80078, Italy; 2 Dip. Multidisciplinare di Specialità Medico-Chirurgiche e Odontoiatriche, Seconda Università di Napoli, via L. De Crecchio 6, Napoli 80138, Italy; E-Mails: fabriziadapuzzo@gmail.com (F.A.); grassiavincenzo@libero.it (V.G.); letizia.perillo@unina2.it (L.P.); 3 Dip. di Medicina Sperimentale, Seconda Università di Napoli, S. Maria di Costantinopoli 16, Napoli 80138, Italy; E-Mail: maria.lepore@unina2.it

**Keywords:** micro-Raman spectroscopy, wavelet data analysis, gingival crevicular fluid (GCF), periodontitis, orthodontic processes

## Abstract

Micro-Raman Spectroscopy is an efficient method for analyzing biological specimens due to its sensitivity to subtle chemical and structural changes. The aim of this study was to use micro-Raman spectroscopy to analyze chemical and structural changes in periodontal ligament after orthodontic force application and in gingival crevicular fluid in presence of periodontal disease. The biopsy of periodontal ligament samples of premolars extracted for orthodontic reasons and the gingival crevicular fluid samples collected by using absorbent paper cones; were analyzed by micro-Raman spectroscopy. Changes of the secondary protein structure related to different times of orthodontic force application were reported; whereas an increase of carotene was revealed in patients affected by periodontal inflammation.

## 1. Introduction

The periodontal ligament (PDL) is a membrane-like connective tissue interposed between the tooth root and the alveolar bone of which the main component is represented by collagen fibers. This tissue has an important role in supporting the tooth in the bone socket of the jaw and also in maintaining homeostasis of the surrounding tissues, such as alveolar bone and cementum [1]. During the early stage of application of orthodontic forces an inflammatory process occur in the periodontium [2]. Among optical diagnostic techniques, micro-Raman Spectroscopy (μ-RS) constitutes an efficient method for analyzing biological specimens due to its sensitivity to subtle chemical and structural changes [3–5]. Our preliminary study, based on biopsy of PDL samples of premolars extracted for orthodontic reasons and analyzed by μ-RS, confirmed its efficacy in evaluate, at a nanometer scale, the macromolecular folding of periodontal fibers after orthodontic force application [6]. In fact, μ-RS showed the secondary protein structure changes related to different times of orthodontic force application, which may be similar in presence of periodontal diseases. Periodontal inflammation is associated with changes in saliva composition and also with an increase in the flow of the gingival crevicular fluid (GCF) into the periodontal space [3,7]. GCF is a fluid deriving from the epithelium lining of the gingival sulcus. The increased GCF flow contributes to host defense by flushing bacterial colonies and their metabolites away from the periodontal space, thus restricting their penetration into the tissue. A previous study [7] used the collection of GCF as a simple and noninvasive diagnostic procedure in humans to evaluate biologically active substances expressed by cells within the periodontium in response to mechanical stimuli by orthodontic tooth movement. The site-specific nature of GCF collection is useful in monitoring the factors related to the periodontal diseases and may therefore be of diagnostic value [8]. Sharma *et al.* [9] found an increasing trend of osteopontin level in GCF comparing health subjects with patients affected by gingivitis or periodontitis. Ngo *et al.* [10] reported a comprehensive proteomic analysis identifying different proteins and peptides into GCF.

The aim of this study was to use μ-RS to analyze chemical and structural changes in PDL and GCF samples.

## 2. Experimental Section

### 2.1. Sample Preparation

#### 2.1.1. PDL Biopsy Samples after Orthodontic Force Appliance

Samples of periodontal ligaments (PDL) of orthodontic patients; aged between 13 and 21 years; treated with extraction of upper and/or lower premolars have been selected. Informed consent was obtained from each minor patient's parents or adult patients after providing them detailed information about the clinical trial. For this study we utilized a 50 gr Sentalloy^®^ (GAC International, Bohemia, NY, USA) coil spring *in vivo.* Orthodontic brackets with power pin (MBT; 3M Unitek; Monrovia; Cali, CO, USA) were applied on the buccal surface of upper and/or lower first premolars; and bands were placed on upper and/or lower first premolar only on the right side. Patients were randomly assigned to 3 groups; of which tooth extractions were performed after 2, 7 or 14 days of force application, respectively. The PDL was scarified from radicular surface on the pressure and tension side of the extracted premolars using a one-way lancet. Each sample has dimensions of the order of few mm^3^. The results were compared with PDL samples of normal homologous teeth (control). The PDL samples were fixed in 4% paraformaldehyde (PFH) at least in 3 h. PFH was removed by centrifugation (2000 rpm for 2 min). To fix samples graded series of ethanol solutions (50%–70%–80%–95%) were used. Samples were leaved in each ethanol's solution for 1 h at room temperature starting from 50% solution to 95% and then they were stored in ethanol 100% until analysis.

#### 2.1.2. GCF Samples

**Figure 1.**
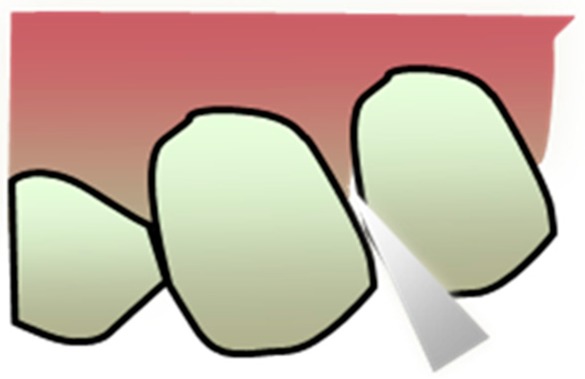
Schematic view of GCF collection method.

Sample of GCF was pooled from informed periodontal and health patients by using standardized sterile absorbent paper cones inserted 1 mm into the gingival crevice and left *in situ* for 30 s, without blood, saliva and plaque contamination (Figure 1). Contamination of the GCF samples was minimized by carefully cleaning the tooth with cotton pellets and drying it by a gentle air stream. The GCF samples were transferred to plastic vials and stored at room temperature until analysis.

### 2.2. Micro-Raman Spectroscopy

Samples were excited by the light of a He-Ne laser operating at a wavelength λ = 633 nm, with a maximum nominal power of 17 mW. The signal was collected by a *Jobin-Yvon TriAx 180* monocromator, equipped with a liquid N_2_ cooled CCD and a grating of 1800 grooves/mm, allowing a spectral resolution of 4 cm^−1^. The laser light was focused on the sample surface by means of a 100 × (n.a. = 0.90) optical objective on an excitation area of about 10 μm of size. The spectra were obtained using accumulation times ranging in 60–300 s.

### 2.3. Data Analysis

#### 2.3.1. Wavelet Based Numerical Data Treatment

The Raman spectra collected from both PDL and GCF samples typically showed a smeared background signal, that may even reach the 80% of the whole average intensity in the case of GCF. In order to enhance the signal readability and attenuate background and noise components, an automatic numerical treatment based on wavelet algorithm was used [11]. The Raman spectrum can be assimilated to a time modulated signal f(*t*) on a finite interval, where the wavenumber shift stands as the variable *t*. The wavelet algorithm cuts up the signal into different “frequency” components, similarly to the conventional Fourier transform, but it uses spatially localized functions with average zero value (namely *wavelets*, small waves) instead of conventional sinusoidal functions, allowing the obtainment of information on both frequency and time dependence. Basically, the signal is represented in terms of the sum of elementary wavelets and decomposed in two signals, one containing the low frequency components (approximation A1) and the other one the fluctuations (detail D1). The algorithm is iteratively applied to the “approximated” part of the function and a higher level of the A2 and D2 component pair is generated. A hierarchical representation of the data set was thus obtained allowing a multi-resolution analysis, known as discrete wavelet transform (DWT) in which details or fluctuations of different level of resolution were represented by the superposition of wavelets with suitable dilation. Starting from the decomposed signal, the spectrum can be reconstructed by an inverse process (IDWT). The removal of low and high frequency components implies a significant attenuation of background and non-correlated noise levels, respectively, of the IDWT reconstructed signal. MATLAB 6.5 program (by MathWorks Inc., Natick, MA, USA) was used for wavelet analysis with wavelet family of biorthogonal functions “bior6.8”. The decomposition of the signal was performed up to the level *n* = 8. Subsequently the signal was reconstructed by employing only detail components from D5 to D6.

#### 2.3.2. Subtraction of Substrate Signal (GCF Samples)

GCF samples components of the Raman spectra due to the paper substrates were removed. μ-RS was performed on pristine paper cone (without GCF) with measurements parameters similar to those used for sampling GCF. After the wavelet based data treatment above described, the Raman spectra of GCF samples were compared to the paper cone spectrum by linear regression analysis. Then, the paper cone Raman spectrum was subtracted from the GCF signal, using regression coefficient as scaling factor for signals.

#### 2.3.3. Deconvolution Procedure

In order to determine the basic vibrational modes that contribute to the Raman signal, the spectra were analyzed in terms of convoluted peak functions by using a best-fit peak-fitting routine of GRAMS/AI (2001, Thermo Scientific™, Waltham, MA, USA) program, which is based on the Levenberg-Marquardt nonlinear least-square method. In particular, the wavenumber band of Amide I (1550–1750 cm^−1^) was considered, where significant variations were expected due to protein configuration changes. A mixed Gaussian and Lorentzian peak shape was used [12]. Peaks constituting the spectrum were manually selected in order to define the starting conditions for the best-fit procedure. The best fit was then performed to determine convolution peaks with optimized intensity, position and width. Its performance was evaluated by means of the λ^2^ parameter.

## 3. Results and Discussion

### 3.1. Periodontal Ligaments

**Figure 2.**
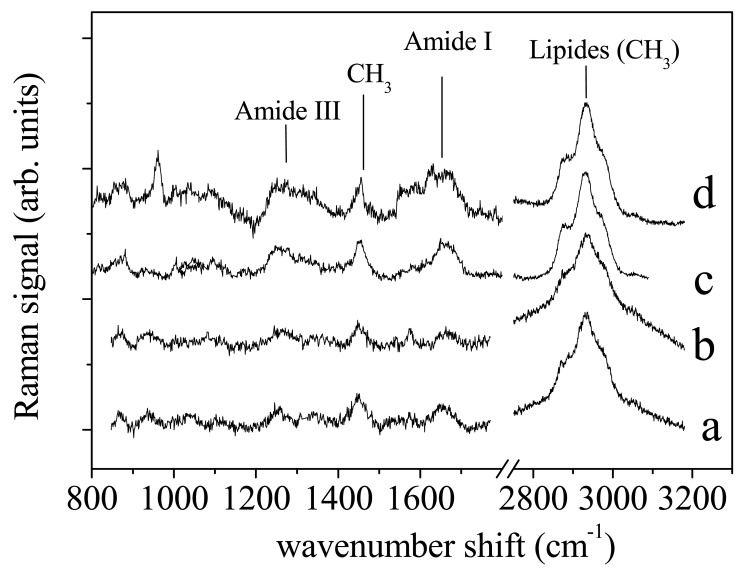
Periodontal ligament micro-Raman spectra for control sample (**a**); and for ligaments after 2 days (**b**); 7 days (**c**) and 14 days (**d**) of orthodontic treatment, respectively.

A representative Raman spectra of PDL was shown in Figure 2a and compared with spectrum of ligaments after 2 days (Figure 2b), 7 days (Figure 2c) and 14 days (Figure 2d) of orthodontic process by Sentalloy coil spring 50 gr applied *in vivo* The samples belonged to the same patient. In both spectra were clearly evinced the main contributions to Raman signal of protein and lipids. Amide I (between 1600 and 1700 cm^−1^) and Amide III (between 1200 and 1300 cm^−1^) mode were the two major bands assigned to protein vibrations. The broad and intense peak centred at 2930 cm^−1^ was assigned to lipids (mainly due to CH_3_ modes). Finally, the peak ad about 1450 cm^−1^ was assigned to CH_2_ scissoring mode from both lipid and protein components. Even if at first glance the two spectra looked similar, some differences occurred and can be pointed out by an accurate analysis of vibrational modes contributing to the Raman signal.

**Figure 3.**
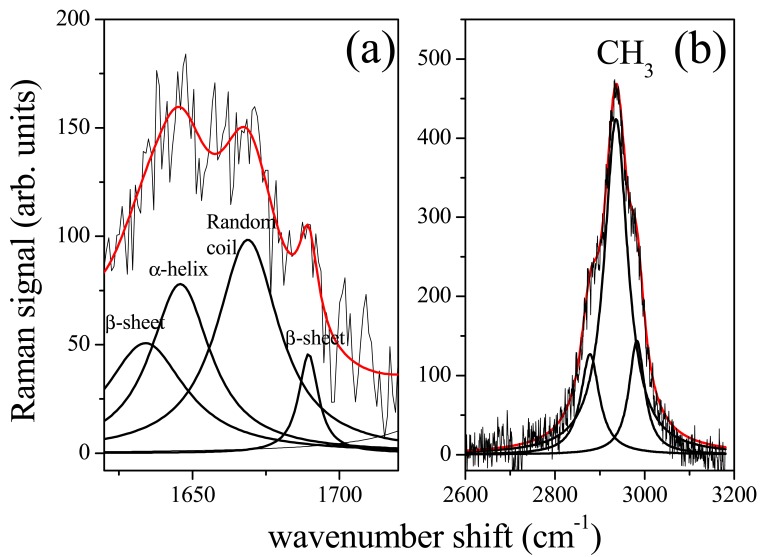
Deconvolution of Raman spectrum of control PDL sample in the Amide I (**a**) and in the lipid CH_3_ (**b**) spectrum region. Red line is the curve obtained by envelop of peaks obtained by the fit procedure.

**Figure 4.**
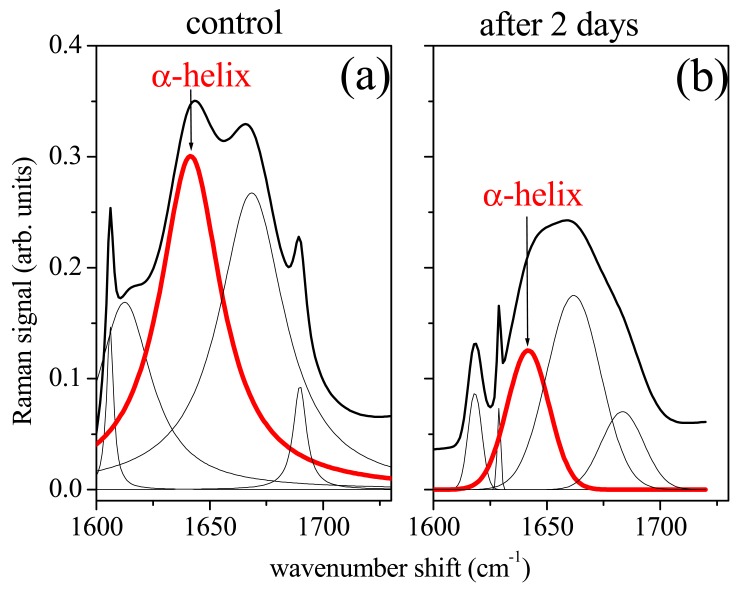

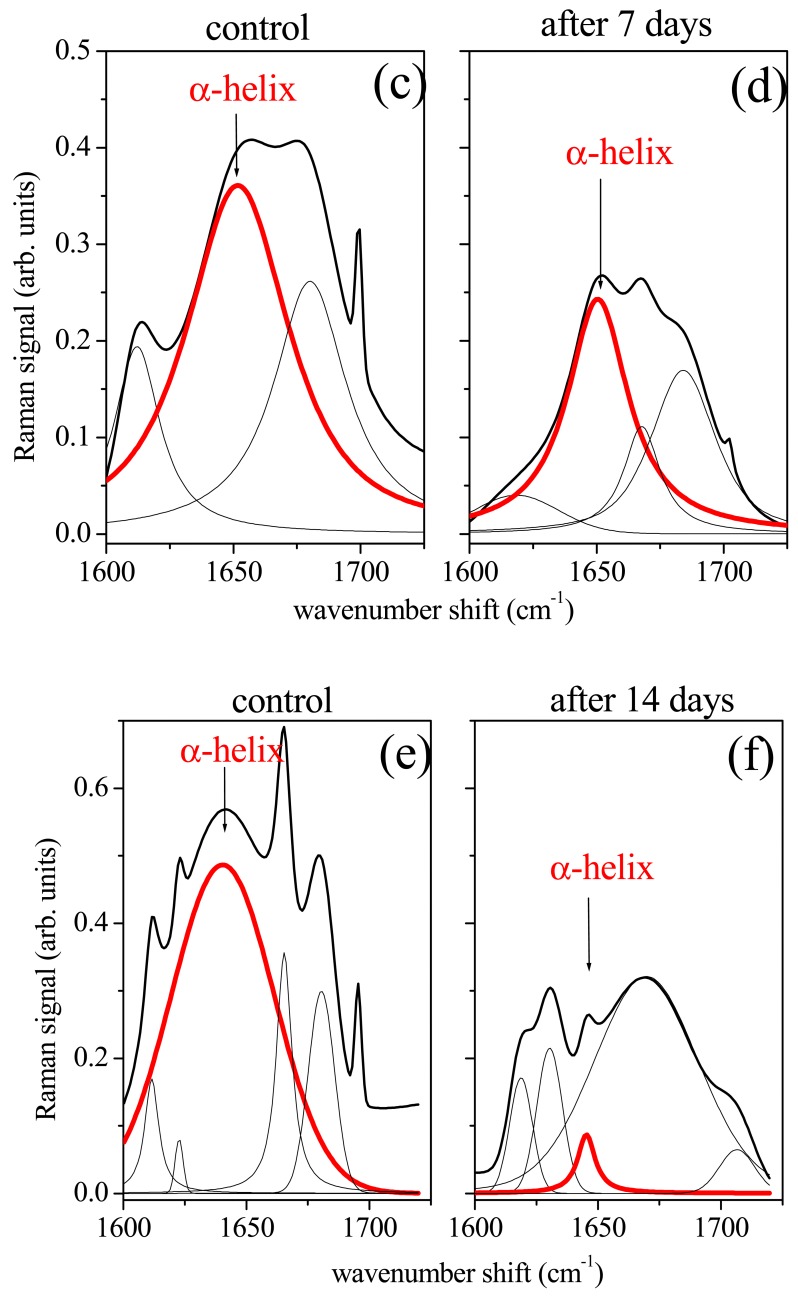
Amide I region of Raman spectrum of control PDLs (**a**,**c**,**e**) and after orthodontic treatment of 2 days (**b**), 7 days (**d**) and 14 days (**f**) respectively. Experimental data have been fitted by a convolution of Lorentzian or Gaussian functions. Raman mode assigned to α-helix is underlined by the red line.

Amide I band consists of a limited number of major components related to the secondary structure of protein [13,14]. The spectrum is dominated by the α-helix mode, centred at about 1645–1650 cm^−1^, with shoulders at ∼1620 cm^−1^, ∼1668 cm^−1^ and ∼1680 cm^−1^ characteristics of β-sheet (or 3_10_-helix), random coil and β-sheet secondary structure conformations, respectively [14]. As an example the deconvolution of Raman signal in the spectrum region of Amide I and CH_3_ lipid band was reported in Figure 3a and Figure 3b respectively for the (a) control sample. To note, a large variations occured in the Amide I spectrum region for (b) sample (Figure 4). The peak deconvolution of Amide I Raman spectra measured on PDL samples extracted from different patients was reported in Figure 4a,c,ef. PDLs were obtained from not orthodontically treated teeth (control samples). Deconvolution exhibited a strong similarity regarding the position and intensity of Raman modes. As reported in Table 1, the modes can be in general modelled by a Lorentzian function, as expected in the case of homogeneous tissue. Gaussian function was instead more suitable to model signal from heterogeneous and disordered tissue [12]. In Figure 4b,d,f was reported the Amide I region Raman spectrum for PDL tissue after 2, 7 and 14 days of orthodontic treatment, respectively.

For each spectrum was shown the deconvolution in terms of mixed Lorentzian/Gaussian functions. For each sample the Raman signal intensity was normalized to the intensity of the Raman peak at 2932 cm^−1^, assuming the lipid component of the tissue to be less sensitive to strain process. In treated samples, peaks assumed in general a Gaussian shape, with exception of α-helix mode which results Gaussian shaped only in the case of two days treatment.

After the orthodontic treatment the intensity of the α-helix mode, and of whole Amide I band, decreased with respect to that one of signal from control samples. With the time length of the orthodontic process the remaining component modes became broader and stronger with respect to α-helix mode. As expected the strain due to the orthodontic process affected the structural properties of tissue of periodontal ligaments. In terms of Raman response, we observed a decrease of the signal in the Amide I region with time duration of the orthodontic process. In particular the mode assigned to α-helix secondary structure decreased of about 75% of its pristine value after 14 days of treatment. The intensity change resulted large in the initial stage of the process (two days) while a little recover seemed to occur in the first week of treatment. The responses to strain forces of helical protein structures were considered in literature both theoretically [15] and experimentally [16]. The helical structure gives to protein an enhanced resistance to mechanical strain, prevents breakage and withstands large deformations. This behavior was explained in Reference [16] by a mechanism of molecular unfolding of the α-helix, through reversible breaking of hydrogen bonds, resulting in the formation of yield regions that allows the dissipation of mechanical energy and avoids breakage of strong molecular bonds. In terms of Raman response we expected a decrease of signal intensity of α-helix component due to a partial transition from α-helix to β-sheet conformations, promoted by hydrogen relocation, and an increase of disorder.

**Figure 5.**
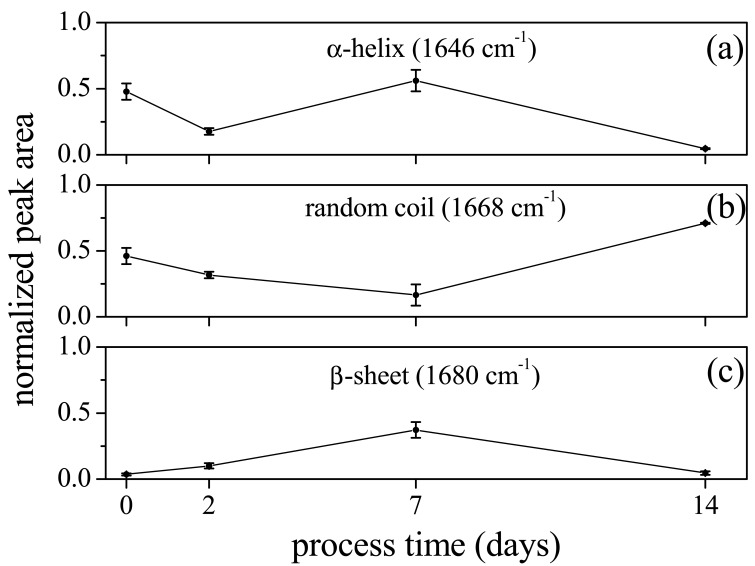
Peak area of subband components of PDL Raman spectrum in the Amide I region, as a function of the orthodontic treatment time, for α-helix (**a**); random coil (**b**); and β-sheet mode (**c**). The data are normalized to the Amide I mode total area.

This effect was experimentally verified in Reference [16], where single keratin fibers, from hair, were monitored by Raman spectroscopy during strain process. In that study, a depression of Raman signal of α-helix band in Amide I and a continuous increase of the β-sheet component with strain strength were, in fact, observed. These results were consistent with the behavior observed in the periodontal ligaments. The orthodontic process induced some modifications in the secondary protein structure of the tissue resulting in a decrease on the intensity of the α-helix Raman mode. This change occurred mainly at the beginning of the process. With time, after some days, a readjustment of the protein structure occurred probably due to a relocation and bond formation of H atoms. For long process time (14 days) some additive mechanisms should occur, probably due to protein denaturation (inflammatory processes) and the β-sheet/random disorder component Raman signal prevails on that one from ordered α-helix component. Together with intensity, the peak shape of Raman signal also changes in treated samples. These Raman peaks can be easily modeled by a Gaussian shape, while spectra of control samples exhibit well-shaped Lorentzian peaks (see Table 1). In general treated sample spectra have wider peaks than those of control ones. In the Amide I region these changes are particularly meaningful because they are related to modifications of the secondary protein structure, as above discussed. In Figure 5 the dependence on the orthodontic process time of the peak area of the main subband modes of Amide I spectrum region is reported. The peak area values of modes assigned to α-helix at 1646 cm^−1^ (Figure 5a), random coil at 1668 cm^−1^ (Figure 5b) and β-sheet at 1680 cm^−1^ (Figure 5c) have been normalized to the total area of Amide I region, in order to evidence the relative contribution of Amide I vibrational modes. The observed features are consistent with the considerations above discussed about the Raman signal intensity dependence on orthodontic process time.

### 3.2. Gingival Crevicular Fluid

**Figure 6.**
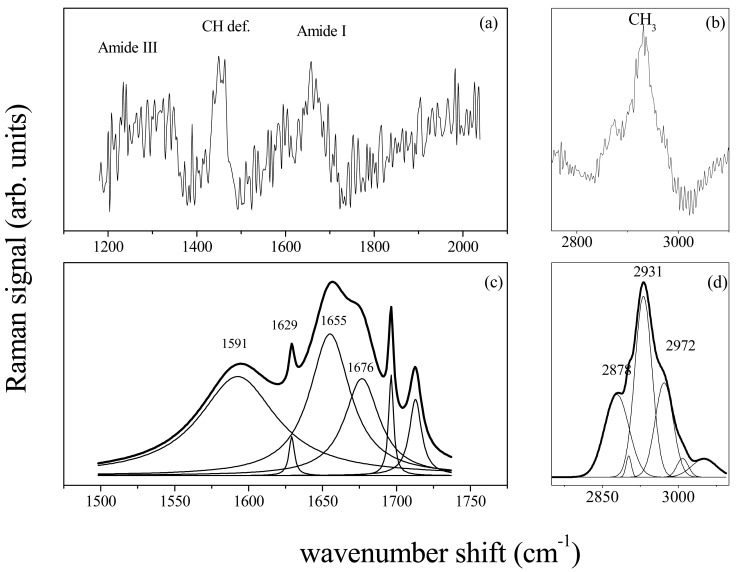
Raman spectrum of GCF from healthy patient for the 1200–2000 cm^−1^ (**a**); and 2500–3100 cm^−1^ wavenumber range (**b**); Deconvolution in Gaussian/Lorentzian peaks of the Amide I spectrum region (1500–1750 cm^−1^) (**c**); and CH_3_ region (2800–3000 cm^−1^) (**d**).

**Figure 7.**
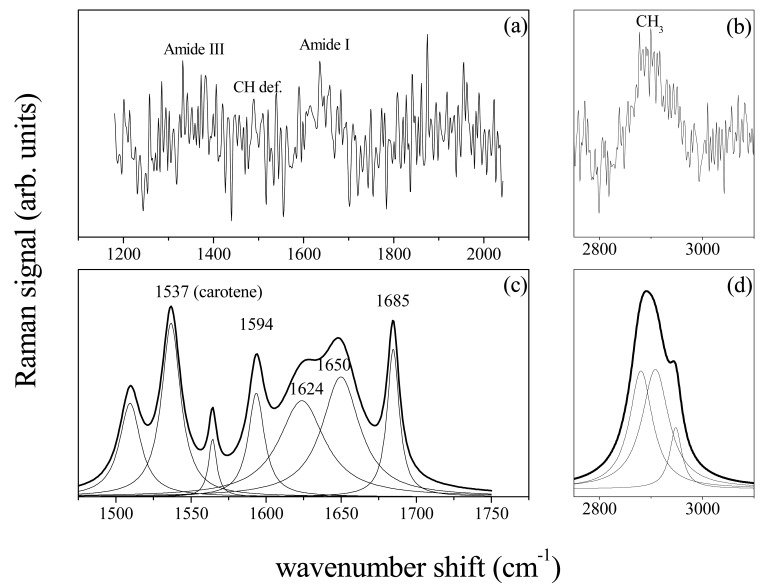
Raman spectrum of GCF from chronic Periodontitis affected patient, for the 1200–2000 cm^−1^ (**a**); and 2500–3100 cm^−1^ wavenumber range (**b**); Deconvolution in Gaussian/Lorentzian peaks of the Amide I spectrum region (1500–1750 cm^−1^) (**c**); and CH_3_ region (2800–3000 cm^−1^) (**d**).

The Raman spectra reported in Figure 6a,b were obtained from a GCF sample of a health patient. The substrate contribution and background signals have been subtracted by using the data treatment previously described. As in the case of PDLs, the Raman signal is characterized by typical vibrational modes of biological tissues and proteins. Peaks assigned to Amide III (1240–1300 cm^−1^), CH deformation modes (1440–1450 cm^−1^) and Amide I (1600–1700 cm^−1^) are clearly discernible in Figure 6a. At higher wavenumbers the lipid and protein CH_3_ bond stretching modes are observed Figure 6b. The deconvolution of the signal in terms of Lorentzian and Gaussian functions is reported in Figure 6c,d for the spectrum region of Amide I and CH_3_ modes, respectively. The secondary protein structures are clearly evinced in the Amide I region (Figure 6c) with the predominant peak of the α-helix mode at 1655 cm^−1^. The spectrum of GCF from a chronic periodontitis affected patient reveals some differences in the secondary protein structure. The spectrum deconvolution for the Amide I region is reported in Figure 7c.

When compared with Figure 6c, it was noticed in Figure 7c an intense peak at about 1537 cm^−1^ which is likely due to the formation of isomerization products containing C=C groups related to an increase of degraded carotene in GCF [17]. Carotenoid concentration in GCF was actually expected to increase with the severity increase of the disease and in chronic periodontitis, with respect to healthy or gingivitis control [3,18,19]. Because of the protein nature of the osteopontin, its presence as expected from Reference [8], cannot be evidenced since its contribution can not be distinguished from that of the other protein components.

## 4. Conclusions

The μ-RS can be useful to monitor chemical and structural changes in PDL and GCF samples.

Raman spectra showed interchain arrangements and macromolecular conformation changes in the periodontal fibers after different times of orthodontic force application and during periodontal diseases. Thus, the potentiality of μ-RS in reporting the specific molecular fingerprinting in medical applications was confirmed although a clinical validation of the proposed methods is required.

## Figures and Tables

**Table 1.** Peak centers (in cm^−1^) of main Raman modes of Amide I region identified by deconvolution of experimental data. Numbers in bold refer to Gaussian functions and normal ones to Lorentzian functions.

**Assignment**	**Control**	**After 48 h**	**Control**	**After 7 Days**	**Control**	**After 14 Days**
β-sheet	1612.5	**1617.8**	1612.1	**1618.4**	1622.7	**1618.8**
?	-	-	-	-		**1630.4**
α-helix	1641.5	**1641.7**	1651.8	1650.5	**1640.4**	1645.3
random coil	1668.5	**1661.8**	-	1667.8	1665.4	**1669.6**
β-sheet	1689.7	**1683.3**	1680.1	**1684.1**	**1680.5**	**1706.5**
